# Potential effect of *Commiphora myrrha* resin on *Eimeria labbeana*-like-induced oxidative stress in *Columba livia* domestica

**DOI:** 10.3389/fcimb.2025.1724123

**Published:** 2025-11-06

**Authors:** Rewaida Abdel-Gaber, Shurug Albasyouni, Simeon Santourlidis, Saleh Al Quraishy, Esam Al-Shaebi

**Affiliations:** 1 Department of Zoology, College of Science, King Saud University, Riyadh, Saudi Arabia; 2 Epigenetics Core Laboratory, Institute of Transplantation Diagnostics and Cell Therapeutics, Medical Faculty, Heinrich-Heine University Düsseldorf, Düsseldorf, Germany

**Keywords:** coccidiosis, pigeons, natural treatment, myrrh resin, oxidative stress, IHC study

## Abstract

**Background:**

*Eimeria* infection in pigeons induces severe oxidative stress in intestinal tissues, disrupting the balance between oxidant and antioxidant systems and leading to cellular and physiological damage. The resin of *Commiphora myrrha* has long been recognized in traditional medicine for its therapeutic potential.

**Purpose:**

This study evaluated the protective effect of methanolic *C. myrrha* resin extract (MYE) against oxidative stress and cellular injury induced by *Eimeria labbeana*-like infection in pigeons.

**Methods:**

Twenty-five pigeons (300–380 g) were divided into five groups (G1–G5). Following infection, birds were treated daily with MYE. On day 8 post-infection, intestinal tissues were collected to assess oxidative stress markers, antioxidant enzyme activities, and inducible nitric oxide synthase (iNOS) expression via immunohistochemistry.

**Results:**

Infection with *E. labbeana*-like markedly elevated intestinal levels of nitric oxide (NO), malondialdehyde (MDA), and hydrogen peroxide (H_2_O_2_), indicating enhanced oxidative stress. MYE administration significantly reduced these markers compared to infected controls. Concurrently, MYE enhanced antioxidant defense by increasing catalase (CAT), superoxide dismutase (SOD), and reduced glutathione (GSH) activities. Moreover, MYE modulated iNOS expression, suggesting regulation of inflammation-associated oxidative pathways.

**Conclusion:**

Methanolic extract of *C. myrrha* resin effectively mitigated oxidative and related intestinal issues induced by *E. labbeana*-like infection in pigeon intestines. These findings highlight its potential as a natural source of antioxidant and anticoccidial agents for managing avian coccidiosis.

## Introduction

Avian coccidiosis is a highly prevalent intestinal disease that poses significant economic challenges to the global poultry industry. This disease is primarily caused by a group of protozoan parasites belonging to the genus *Eimeria* (Quiroz-Castañeda and Dantán-González, 2015). Transmission occurs through the ingestion of sporulated oocysts—the infective stage of the parasite—shed in the feces of infected birds. Once ingested, the oocysts release sporozoites that invade intestinal epithelial cells, initiating a complex lifecycle characterized by multiple replication phases. This invasion leads to extensive intestinal damage, impaired nutrient absorption, and pronounced oxidative stress (McDougald, 2013; Tompkins et al., 2023). Among various bird species, pigeons are notably affected by *E. columbae*, *E. columbarum*, *E. labbeana*, and *E. labbeana*-like, each showing varying degrees of pathogenicity (Yang et al., 2016).

Anticoccidial drugs play a pivotal role in the management of *Eimeria* parasites, which are the causative agents of coccidiosis in livestock and poultry (Zhao et al., 2024). Among the most prominent classes of these medications are ionophores, including well-known compounds such as monensin and salinomycin (D’Alessandro et al., 2015). These ionophores operate by manipulating the ion balance within the parasite, disrupting essential processes necessary for its growth and reproduction. Similarly, vitamin antagonists like amprolium interfere with the metabolic pathways of the parasite by mimicking vital vitamins, thereby inhibiting its ability to thrive within the host (Lebkowska-Wieruszewska and Kowalski, 2010). Quinolones, such as diclazuril, target specific stages of the *Eimeria* lifecycle, effectively crippling the parasite at critical developmental points (Gadelhaq et al., 2017). The design and application of these anticoccidial agents aim to minimize the survival and reproductive capacity of *Eimeria*, thus providing a therapeutic advantage in controlling the infections they cause. By carefully targeting various stages of the parasite’s lifecycle, these drugs significantly reduce the incidence of coccidiosis symptoms in infected animals.

The extensive and often negligent use of these medications in agricultural practices has led to significant challenges, primarily the emergence and spread of drug resistance among *Eimeria* populations. This phenomenon arises when parasites develop genetic adaptations that allow them to resist the medical effects of treatments, thereby reducing their effectiveness over time. The grim consequence of such resistance is an increased difficulty in managing coccidiosis outbreaks, as traditional treatment options become less effective. Additionally, Bafundo et al. (2008) emphasize that the rising resistance makes it increasingly complex for veterinary practitioners and farmers to maintain control over *Eimeria-*specific infections. As resistant strains proliferate, the reliance on existing drugs may inadvertently contribute to a cycle of ineffective treatment, underscoring the urgent need for responsible and strategic use of anticoccidial therapies in livestock management. The ongoing research and development of new drugs aims to address resistance issues and protect animal health. Plants and their active phytochemicals provide several advantages, including minimal drug residues and side effects, a lower risk of developing resistance, and reduced costs. As a result, they are promising options for anticoccidial treatments (Lillehoj et al., 2018).

The *C. myrrha* (myrrh) has a rich history of traditional medicine use, particularly in Saudi Arabia, where it is valued for its therapeutic benefits (Bakhotmah and Alzahrani, 2010). The chemical composition of myrrh resin is intricate, containing numerous bioactive compounds such as triterpenoids, diterpenoids, steroids, and lignans, all of which contribute to its medicinal properties (Alabdalall, 2024). Research has uncovered a range of health-promoting effects associated with myrrh. It exhibits hypotensive properties that can aid in lowering blood pressure (Batiha et al., 2023), demonstrates anticancer effects by potentially inhibiting tumor growth (Sun et al., 2020), and is recognized for its powerful antioxidant capabilities that protect against cellular damage (Ashry et al., 2010). Furthermore, myrrh possesses antimicrobial properties that combat various pathogens (Kuete et al., 2012), antirheumatic effects that may relieve joint pain (Su et al., 2015), lipid-lowering qualities that support heart health (Omer and Al-Dogmi, 2018), and anti-inflammatory actions that reduce inflammation throughout the body (Akbar, 2020). More recently, myrrh has been investigated for its potential in treating coccidiosis, a parasitic disease affecting birds. An *in vivo* study conducted by Albasyouni et al. (2024a) revealed that extracts derived from myrrh can significantly enhance intestinal health in pigeon models, effectively curbing the proliferation of *Eimeria* parasites, which are responsible for this affliction. Additionally, *in vitro* studies have demonstrated myrrh’s efficacy against *E. labbeana*-like species in pigeons (Albasyouni et al., 2024b). Despite these promising findings, there remains a scarcity of evidence regarding myrrh’s oocysticidal effects in pigeons that have been infected with *Eimeria*, highlighting an area that warrants further research.

Despite these promising findings, the precise mechanisms by which *C. myrrha* exerts its protective effects against *E. labbeana*-like infection remain poorly understood, particularly regarding its modulation of oxidative stress and inflammation in pigeon intestinal tissues. This research addresses this gap by investigating the effects of methanolic *C. myrrha* resin extract on oxidative stress biomarkers, antioxidant enzyme activities, and inducible nitric oxide synthase (*iNOS*) expression during *E. labbeana*-like infection in pigeons. By doing so, this study provides novel mechanistic insights into the potential use of *C. myrrha* as a natural anticoccidial and antioxidant agent, contributing to the development of safer alternatives for avian coccidiosis management.

## Materials and methods

### Ethical consent

The study protocol received approval from the Research Ethics Committee (REC) for Laboratory Animal Care at King Saud University (Riyadh, Saudi Arabia), with registration number KSU-SU-23-45. All procedures were conducted in full accordance with applicable laws.

### Plant collection and extract preparation

The resin of *C. myrrha* (myrrh) was obtained for this study from a local market in Riyadh, Saudi Arabia. The specimen was carefully verified at the herbarium of the Botany Department at King Saud University to ensure correct identification and classification. To keep proper records and support future research, these specimens were given a unique voucher number, KSU-23539. For comparison in the study, amprolium (AMP) (Amproxine 20%, water-soluble powder) was purchased from Gulf Veterinary Pharmacy in Riyadh, Saudi Arabia, and used as a standard anticoccidial reference to assess the properties of myrrh.

Following the suggested methodology of Akande et al. (2021), 100 grams of myrrh resin were crushed into powder using a Hummer Grinder (Edison Electric, ED-CG1400, China), ensuring a uniform consistency. Approximately 100 grams of this powdered sample was placed in a container, where it was gently agitated while being immersed in 1,000 milliliters of 70% methanol, as this solvent system effectively extracts both polar and moderately nonpolar phytochemicals, including phenolics and terpenoids, which contribute to the antioxidant and therapeutic properties of *C. myrrha*. This percolation process lasted for a full 24 hours at room temperature, facilitating the extraction of soluble compounds from myrrh. After the extraction period, the mixture was carefully strained using Whatman filter paper No. 1, which effectively separated the liquid extract from the solid residue. The resulting filtrate was then subjected to a rotary vacuum evaporator, specifically a Buchi model from Switzerland, where it was dried at a controlled temperature of 45°C. This process concentrated the extract, leaving behind a potent methanolic crude myrrh extract (MYE), which was subsequently diluted with distilled water in a weight-to-volume ratio to prepare the desired dosage for further use.

### Parasite strain and preparation

The *E. labbeana*-like strain used in this study was obtained from naturally infected domestic pigeons (*Columba livia domestica*) collected from local poultry farms in Riyadh, Saudi Arabia. The oocysts were isolated from fecal samples, purified, and sporulated in 2.5% (w/v) potassium dichromate (K_2_Cr_2_O_7_) at 24°C for 24–36 hours (El-Ashram and Suo, 2017). Morphological examination and measurement of the sporulated oocysts were performed according to standard taxonomic keys to confirm their identity as *E. labbeana*-like, following the descriptions of Yang et al. (2016). The purified strain was maintained under laboratory conditions until use in experimental infection.

Five pigeons were employed to propagate *Eimeria* oocysts. Qudoos et al. (2020) stated that 3×10^4^ sporulated *Eimeria* oocysts were orally inoculated into each pigeon. Oocysts were detected in the infected pigeons’ feces eight days post-infection (p.i.). The *Eimeria* oocysts were recovered and allowed to sporulate as mentioned above. After centrifuging the sporulated *Eimeria* oocysts in phosphate-buffered saline (PBS) for 5 minutes at 2500 rpm, they were washed three times with distilled water.

### Pigeons, housing, and experimental design

A total of 25 white domestic pigeons (*C. livia domestica*), weighing 300–380 g, were obtained from the local animal market in Riyadh, Saudi Arabia. The pigeons were acclimated for one week and kept under standard conditions, including a 12-hour light/dark cycle and a temperature of 23 ± 5°C. They had unlimited access to tap water and a balanced seed diet. The pigeons were housed at the animal facility of the Department of Zoology, College of Science, King Saud University, Riyadh (Saudi Arabia).

**Figure 1:**
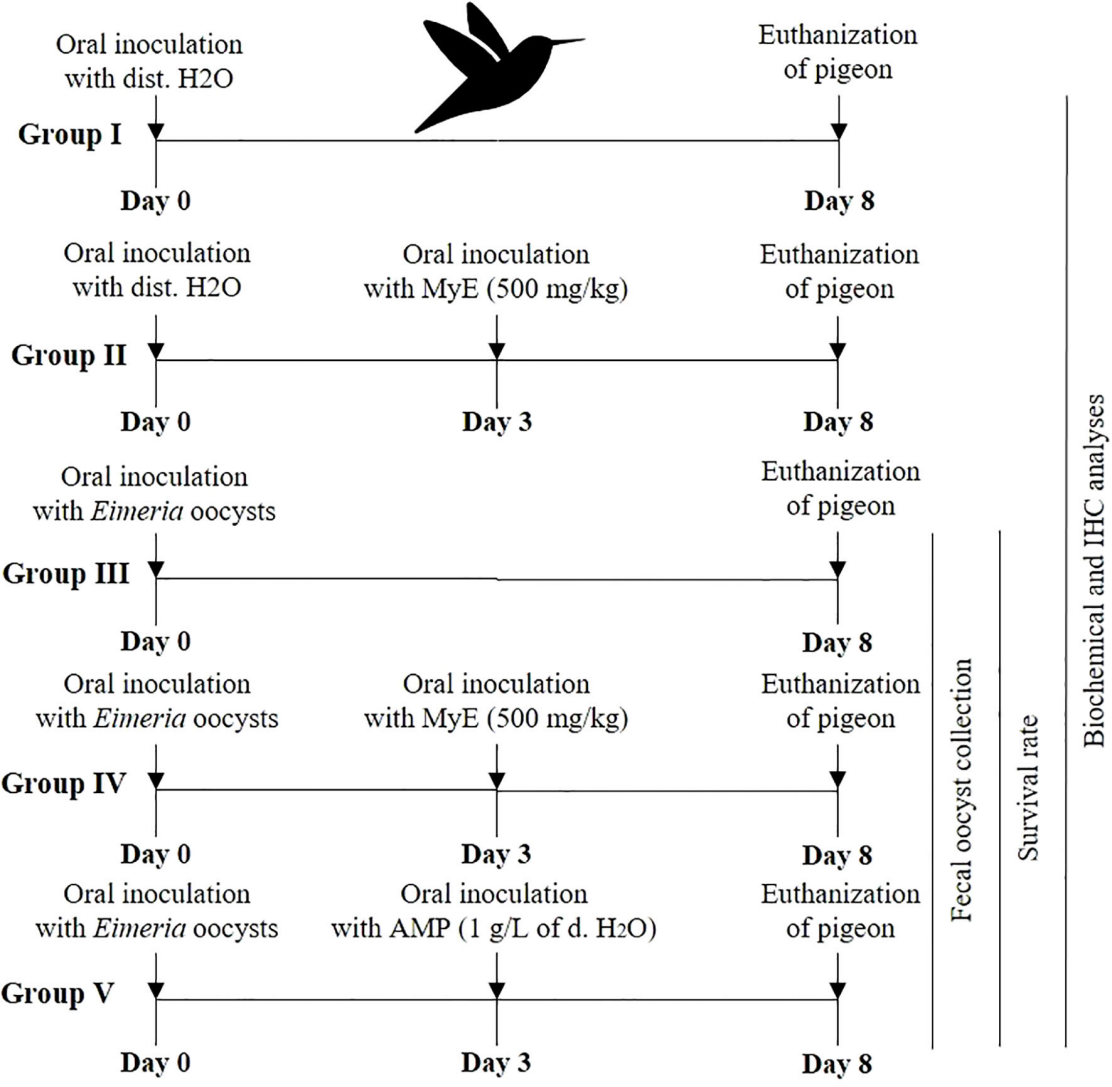
Diagram illustrating the study’s experimental design. (MYE, myrrh extract; AMP, amprolium).

Pigeons were randomly allocated to five experimental groups, each consisting of five birds, as the experimental design in **Figure 1**. Outcome assessments, including oocyst counts, biochemical analyses, and histopathology, were performed by investigators blinded to group assignments whenever feasible. Faecal samples were collected from each pigeon, starting from day 3 p.i. until the end of the experiment, to monitor the oocyst count. At the peak of oocyst count, which occurred on day eight p.i., treatment was terminated. Oocysts were counted as oocysts per gram of feces using the McMaster counting technique, as described by Long et al. (1976).

### Sample collection

On the final day of the experiment, the pigeons were humanely euthanized by administering an overdose of sodium pentobarbital (100 mg/kg; Sigma-Aldrich, St. Louis, MO, USA) via intravenous injection. Following this, a dissection was performed to collect the small intestine. These samples were preserved as follows: (i) some intestinal tissues were immersed in 10% neutral buffered formalin (NBF; Thermo Fisher Scientific, Waltham, MA, USA) for immunohistochemistry (IHC) studies, and (ii) additional intestinal samples were placed in small tubes and stored at -80°C for oxidative status.

### Biochemical analysis

Intestinal tissue was carefully homogenized to a 10% (w/v) concentration using ice-cold 0.1 M phosphate-buffered solution (PBS, pH 7.4) (Albasyouni et al., 2024a). After homogenization, the mixture was centrifuged at 3000 rpm for 15 minutes at 4°C. This step separated cellular debris, leaving a supernatant that was stored at -20°C for later analysis. Oxidative markers in the supernatant were measured using specific diagnostic kits from Bio-Diagnostic Co. (Egypt). The following parameters were measured using their respective standardized protocols: Catalase (CAT, cat. no. CA 25 17) according to Aebi (1984); reduced Glutathione (GSH, cat. no. GR 25 11) following Ellman (1959); Superoxide Dismutase (SOD, cat. no. SD 25 21) based on Nishikimi et al. (1972); Nitric Oxide (NO, cat. no. NO 25 33) as described by Green et al. (1982); Hydrogen Peroxide (H_2_O_2_, cat. no. HP 25) per Aebi (1984); and Malondialdehyde (MDA, cat. no. MD 25 29) according to Ohkawa et al. (1979). Absorbance was measured with Spectra MAX 190, and data were analyzed using SoftMax^®^ Pro software version 6.3.1 for accurate and reliable quantification.

### Immunohistochemical staining of inducible nitric oxide synthase

The fixed intestinal tissues were dehydrated in graded ethyl alcohol, treated with xylene, embedded in paraffin wax, and finally cut into 5 µm sections using a microtome. The sections prepared for IHC labeling were deparaffinized in xylene, rehydrated, and endogenous peroxidase activity was blocked by incubation with 3% H_2_O_2_ for 5 minutes. Sections were then pre-incubated for 30 minutes with a normal serum buffer solution (Diagnostic BioSystems, Serpentine, CA, USA) and incubated for 3 hours at 4°C with a 1:300 dilution of anti-iNOS antibodies (Santa Cruz Biotechnology, CA, USA). This was followed by application of a biotinylated secondary antibody and streptavidin-conjugated horseradish peroxidase (Vision Biosystems Novocastra, Novocastra Laboratories Ltd., Newcastle, UK), prepared according to the kit instructions. The sections were then incubated with 3,3’-diaminobenzidine hydrochloride (DAB) chromogen substrate (Vision Biosystems Novocastra) following the manufacturer’s instructions and counterstained with H&E (Sigma Chemical Co.). Thorough washes with an immunowash buffer (Vision Biosystems Novocastra) were performed between steps. Sections were dehydrated through a graded ethanol series, cleared in xylene, and covered with a thin glass coverslip. All sections were examined for the marker iNOS and photographed using an Olympus BX61 microscope (Tokyo, Japan).

### Statistical analysis

The results of the experiments were presented as the mean value with the standard deviation (SD) for each group. A one-way analysis of variance (ANOVA) was performed to evaluate differences between groups, followed by the Tukey *post hoc* test for additional data analysis. Values with a P-value of 0.05 or less were considered statistically significant.

## Results

**Figure 2:**
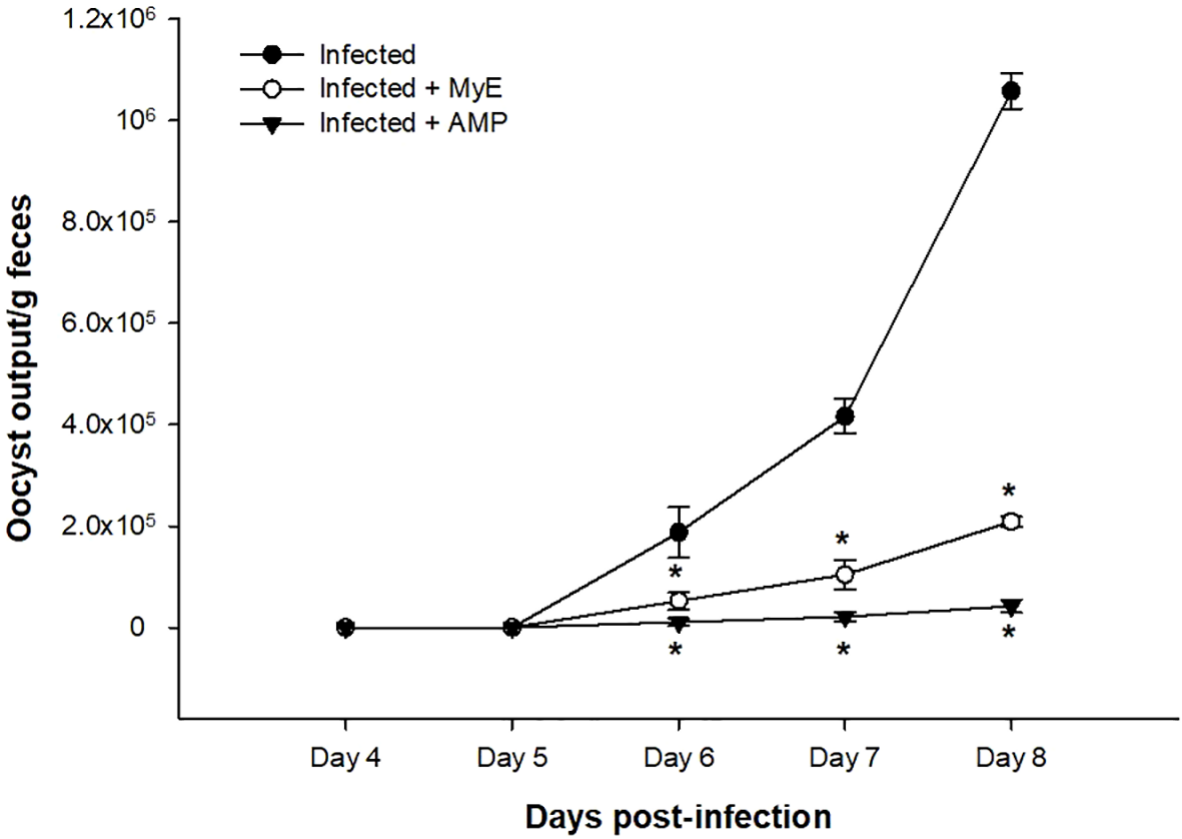
Effect of MYE and AMP treatments on oocyst output in infected groups. Data represent oocyst counts per gram of feces recorded from day 4 to day 8 post-infection. Values are expressed as mean ± SEM (n = 5). ^*^p ≤ 0.05, significant change with respect to the infected group. (MYE, myrrh extract; AMP, amprolium).

**Figure 3:**
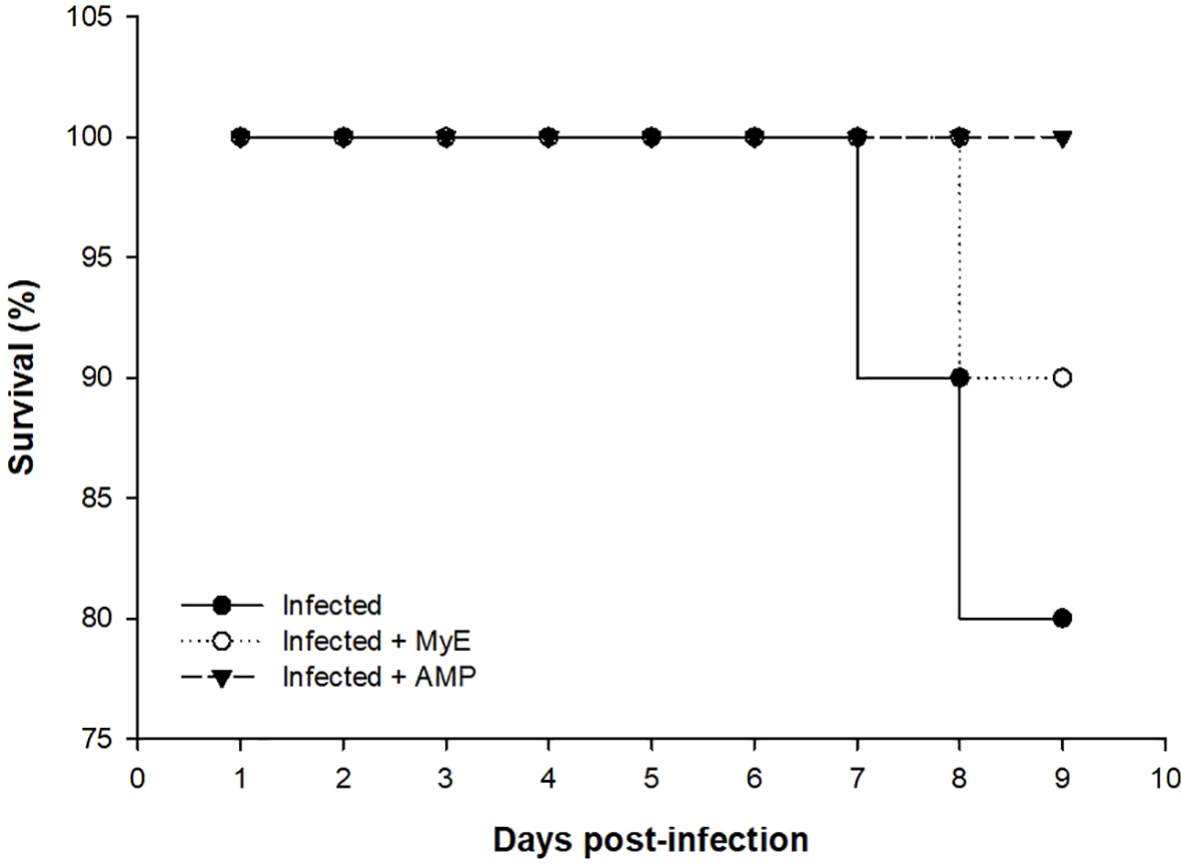
Chart showing the survival (%) of the infected and treated pigeon groups throughout the experimental study. (MYE, myrrh extract; AMP, amprolium).

Fecal *Eimeria* oocyst shedding was highest in the infected group from days 4–8 post-infection (p.i.), whereas administration of both MYE and AMP markedly reduced oocyst counts throughout the observation period (P < 0.001, **Figure 2**). Survival analysis indicated that mortality began on day 7 p.i. in the infected group. MYE treatment extended survival to day 8, improving clinical outcomes, while AMP treatment maintained 100% survival throughout the experiment (P < 0.01, **Figure 3**).

**Figure 4:**
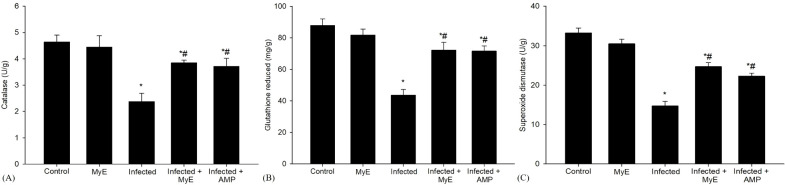
Effects of MYE and AMP treatments on antioxidant enzyme activities in different groups. **(A)** catalase (U/g), **(B)** glutathione reduced (mg/g), and **(C)** superoxide dismutase (U/g). Data are expressed as mean ± SEM (n *=* 5). ^*^p ≤ 0.05, significant change with respect to control, ^#^p ≤ 0.05, significant change with respect to infected group. (MYE, myrrh extract; AMP, amprolium).

Antioxidant enzymes such as CAT and SOD were analyzed on the 8^th^ day after infection (**Figure 4**). The CAT levels were significantly reduced in the infected group (P< 0.001) compared to control pigeons. This notable decrease emphasizes the effect of infection on enzymatic activity. In contrast, when pigeons were treated with MyE, there was a marked improvement in CAT levels (P< 0.01). Additionally, pigeons that received AMP showed a rise in CAT levels (P< 0.01).

Furthermore, the activity level of SOD, an important antioxidant enzyme, was significantly decreased in the infected group (P< 0.001, **Figure 4**) compared to the control group. This decline indicates a notable impairment in the antioxidant defense mechanism in the infected group. In contrast, pigeons treated with MyE and AMP showed a significant recovery in SOD levels (P < 0.01).

The concentration of GSH, a vital antioxidant in biological systems, significantly decreased in the infected group (P< 0.001, **Figure 4**) compared to the control group. This noteworthy reduction suggests that the infection may have impaired the antioxidant capacity of the pigeons’ tissues. After administering treatment, there was a marked increase in GSH levels among the pigeons that received either MyE or AMP (P< 0.01, **Figure 4**), a medication commonly used to prevent and treat coccidiosis.

**Figure 5:**
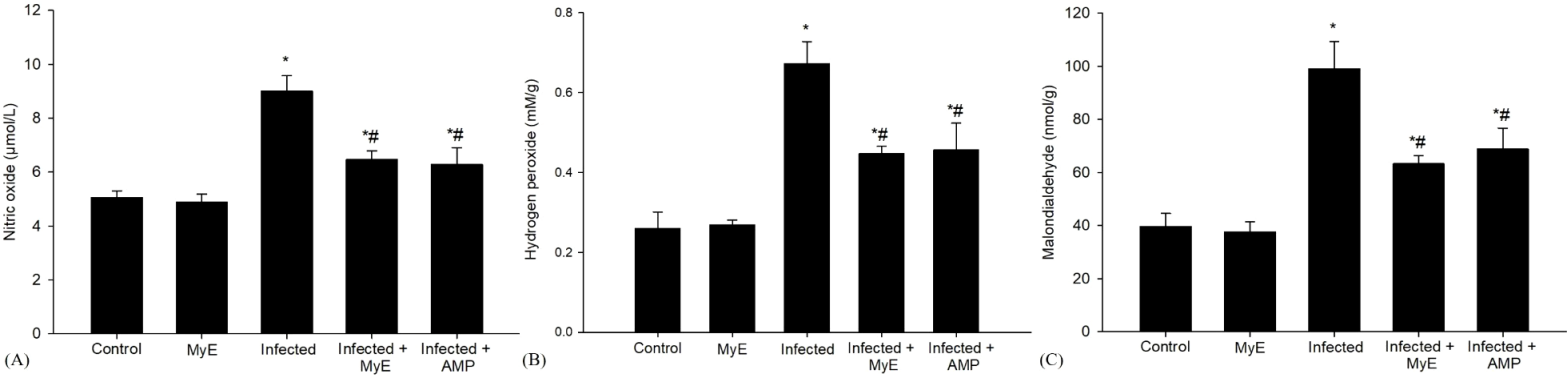
Effects of MYE and AMP treatments on oxidative stress biomarkers in different groups. **(A)** Nitric oxide (µmol/L), **(B)** Hydrogen peroxide (mM/g), and **(C)** Malondialdehyde (nmol/g). Data are expressed as mean ± SEM (n = 5). ^*^p ≤ 0.05, significant change with respect to control, ^#^p ≤ 0.05, significant change with respect to infected group. (MYE, myrrh extract; AMP, amprolium).

Furthermore, the administration of the *Eimeria* parasite caused a significant increase in NO levels (P< 0.001, **Figure 5**). This rise is notable compared to the control group. NO is essential in various physiological processes, including stopping lipid peroxidation, a reaction that can damage cells if unchecked. Following this, the administration of MyE, along with AMP, showed a significant effect on NO levels (P< 0.01, **Figure 5**).

Oxidative stress markers such as MDA and H_2_O_2_ were analyzed on the 8^th^ day after infection (**Figure 5**). The level of MDA, a key indicator of the peroxidation of polyunsaturated fatty acids within cells, showed a significant increase in the infected group exposed to *E. labbeana*-like infection (P< 0.001). This substantial rise indicates a strong oxidative stress response caused by the infection. Conversely, in pigeons treated with the medicinal extract MyE and AMP, there was a notable reduction in MDA levels (P< 0.01).

Compared to the control group, infection with *Eimeria* caused significant cellular damage, marked by a notable increase in reactive oxygen species (ROS), particularly H_2_O_2_, indicating strong oxidative stress within the cells (P< 0.001, **Figure 5**). To assess potential protective effects, pigeons received two different treatments: MyE and AMP. Both treatments significantly reduced oxidative damage caused by the infection induced by *E. labbeana*-like (P< 0.01).

**Figure 6:**
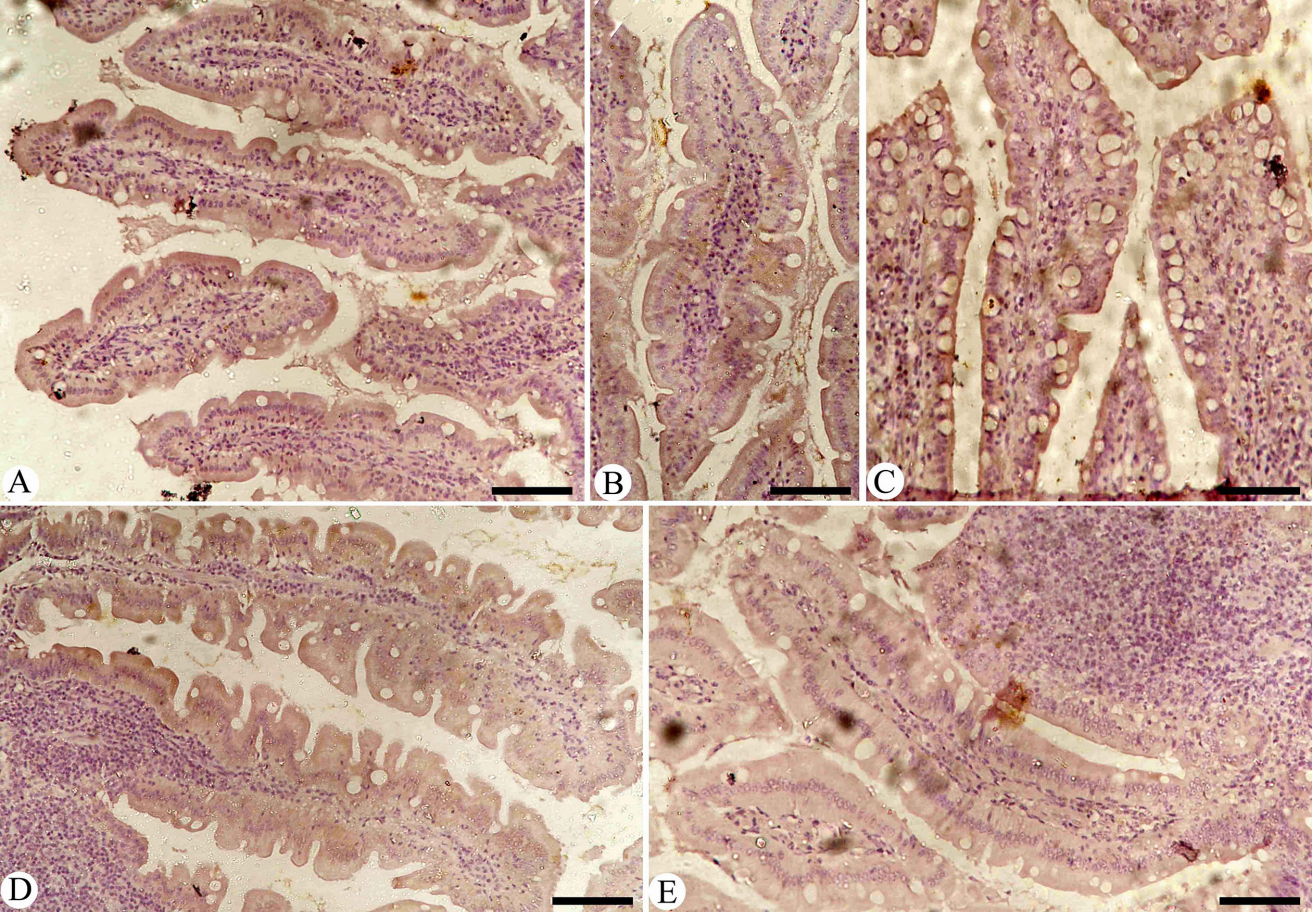
Immunohistochemical detection of iNOs in the pigeon small intestine. **(A)** Control group. **(B)** Non-infected pigeons treated with MyE. **(C)** Infected group. **(D)** Infected pigeons treated with MyE. **(E)** Infected pigeons treated with amprolium. Scale Bar = 100 µm. (MYE, myrrh extract; AMP, amprolium).

Intestinal sections from various experimental groups were stained to assess iNOs expression (**Figure 6**). The results showed a significant increase in iNOs levels in the *Eimeria*-infected group, characterized by many positively stained cells, which sharply contrasted with the baseline levels seen in the control group of uninfected pigeons (P< 0.001). The rise in iNOs expression can be linked to immune and inflammatory responses, especially those driven by the pro-inflammatory cytokine interferon-gamma (IFN-γ). Notably, the spontaneous increase in NO production is closely related to the dysregulated iNOs expression observed during the infection with *Eimeria*, indicating a connection between disease processes and NO synthesis. Further analysis revealed significant changes in iNOs expression after treatment (P< 0.01), with both the infected-treated MyE group and the infected-treated AMP group showing notable differences compared to the untreated infected group.

## Discussion

Coccidiosis remains a major challenge in veterinary health and is one of the leading causes of economic losses and mortality in poultry and pigeon farming, particularly in regions where preventive and treatment options are limited (Mathis et al., 2024). Intestinal coccidiosis is a severe complication caused by infection with various *Eimeria* species. In experimental models, closely related *Eimeria* strains are often used to study host–parasite interactions and evaluate anticoccidial strategies (Zhao et al., 2024). Recently, there has been growing interest in alternative treatments for coccidiosis, especially herbal preparations with antioxidant properties. Several studies have reported anticoccidial effects of different medicinal plants against *Eimeria* species infections in pigeons (Ali et al., 2015; Arafa et al., 2020; Mahmoud et al., 2021; Albasyouni et al., 2024a). In this study, we aimed to assess both the anticoccidial and antioxidant properties of a specific herbal extract, MYE, using an *in vivo* pigeon model infected by amprolium-sensitive strains of *E. labbeana*-like. Our results align with previous reports of Abdel-Gaber et al. (2023) regarding the clinical symptoms and disease severity in coccidiosis-infected pigeons. The anticoccidial efficacy of MYE was demonstrated by a reduction in oocyst output in treated pigeons, likely due to the presence of bioactive compounds in the extract, consistent with findings reported by Albasyouni et al. (2024a; 2024b). These results highlight the potential of herbal medicines as viable alternatives for managing coccidiosis.

Living organisms consistently produce reactive oxygen species (ROS) as a byproduct of cellular metabolism, which is essential for maintaining normal physiological functions. However, environmental factors and certain chemical exposures can lead to excessive ROS accumulation, resulting in tissue injury (Xie et al., 2019). In coccidiosis, *Eimeria* parasites induce high levels of ROS, exacerbating intestinal epithelial damage, lipid peroxidation, and inflammatory responses (Estevez, 2015). Recent studies also demonstrate that coccidial infection disrupts the host antioxidant defenses, causing oxidative imbalance and intestinal tissue damage (Li et al., 2022; Elmahallawy et al., 2022). These ROS also activate pro-inflammatory signaling cascades, including NF-κB and MAPK pathways, which regulate the expression of cytokines and inducible nitric oxide synthase (iNOS) (Surh et al., 2001; Kim et al., 1999). These findings highlight oxidative stress as a central pathological mechanism in coccidiosis and support exploring antioxidant-based therapies to reduce disease severity. Our study indicates that treatment with MYE protects intestinal tissue against oxidative stress during infection with *Eimeria*. This protective effect suggests that MYE supplementation not only mitigates oxidative damage but also enhances the host antioxidant defense system under infection-induced stress. These observations are consistent with recent findings by Albasyouni et al. (2024a), reinforcing the potential antioxidant strategies in managing coccidiosis and its associated oxidative complications.

Antioxidant enzymes such as SOD and CAT play central roles in the body’s defense against reactive oxygen species (ROS), maintaining cellular stability and protecting against oxidative damage (Kurutas, 2016; Bacou et al., 2021). When their levels are imbalanced, however, these enzymes can contribute to oxidative damage of proteins, lipids, and DNA (Tompkins et al., 2023). During *E. labbeana*-like infection, SOD and CAT activities are often significantly decreased in intestinal and other tissues compared to healthy controls. Previous studies support this observation; for instance, Mengistu et al. (2021) reported that reductions in plasma SOD activity are associated with ROS generated during coccidiosis-related tissue damage. CAT activity, however, shows more variability. Some studies, such as Georgieva et al. (2011), observed increased CAT activity in infected birds, likely as a compensatory response to oxidative stress, whereas other studies report reduced CAT activity depending on *Eimeria* species, tissue type, and timing of measurement (Khatlab et al., 2019; El-Ghareeb et al., 2023; Razavi et al., 2024). Mechanistically, superoxide anion radicals (O2^−^), a primary ROS, can inhibit CAT by converting it into inactive ferro-oxy (Fe^3+^) and ferryl (Fe^2+^) states, impairing H2O2 breakdown and increasing oxidative stress (Kono and Fridovich, 1982; Shimizu et al., 1984). In the context of elevated oxidative activity during *E. labbeana*-like infections, such as impaired CAT function, can exacerbate H2O2-mediated tissue damage. Interestingly, treatment with MYE significantly improved intestinal antioxidant defenses in infected pigeons by enhancing natural systems such as SOD and CAT. This effect is likely mediated by polyphenols and flavonoids in MYE, which not only scavenge ROS but may also modulate NF-κB and MAPK signaling to enhance endogenous antioxidant defenses. These findings align with previous reports demonstrating MYE’s antioxidant properties in reducing oxidative damage associated with *Eimeria* species infections (Marcotullio et al., 2011; El-Ghareeb et al., 2023; Albasyouni et al., 2024a).

This study demonstrated that infection with *E. labbeana*-like significantly reduces GSH levels in the intestinal tissue of affected pigeons, indicating compromised antioxidant protection. Previous research supports this observation, showing that *Eimeria* species infections impair the body’s antioxidant defenses, including GSH and related enzyme activities (Santos et al., 2020). Similar decreases in GSH and antioxidant enzymes have also been reported in broiler chickens and other avian models following *Eimeria* challenges (Arabkhazaeli et al., 2011). MYE restored GSH levels, consistent with its flavonoid- and polyphenol-rich composition, which can both directly scavenge ROS and influence redox-sensitive transcription factors, including NF-κB, contributing to tissue protection. Studies have shown that the high phenolic and flavonoid content of MYE contributes to strong antioxidant activity, supporting restoration of GSH levels and tissue protection (Daradka et al., 2021; Lebda et al., 2021; Tsiouris et al., 2021). Protective effects of polyphenol-rich extracts against oxidative stress in coccidiosis have also been documented using pomegranate peel, green tea catechins, and curcumin (Allen, 1997; Idris et al., 2017). Collectively, these findings, along with evidence from El-Ghareeb et al. (2023) and Tchodo et al. (2024), indicate that antioxidant treatments—particularly those rich in flavonoids and polyphenols—can enhance GSH levels, strengthen the overall antioxidant defense system, and reduce oxidative stress associated with parasitic infections. This highlights the potential of antioxidant-rich interventions to restore redox balance and protect intestinal tissue from *Eimeria*-induced oxidative damage.

Nitric oxide (NO) is a critical signaling molecule produced by activated macrophages, with cytotoxic effects against various pathogens (Allen, 1997). In this study, pigeons infected with *Eimeria* exhibited significantly increased NO production in intestinal tissues, indicating its involvement in the host immune response to parasitic infection. Previous research has highlighted the role of iNOS in generating excess NO during coccidiosis. For instance, Allen (1997) reported dose-dependent increases in plasma NO metabolites in chickens infected with *E. tenella*, with peak levels between days 5 and 7 post-infection. Inhibiting iNOS with aminoguanidine reduced hemorrhagic damage, suggesting that NO contributes both to tissue injury and host defense. Similarly, Pirali Kheirabadi et al. (2010) observed elevated serum nitrite and nitrate levels at 10- and 14-day post-infection in birds exposed to multiple *Eimeria* species. Cytokine-mediated immune regulation also plays a role in NO production. Shanmugasundaram et al. (2024) demonstrated that interferon-γ (IFN-γ) stimulates iNOS expression in macrophages during parasitic infection with *Eimeria* in broilers. In pigeons, Salem et al. (2022) observed high intestinal NO levels in untreated infected birds, which gradually declined with treatment over 5–15 days, approaching baseline levels seen in controls. Our findings indicate that MYE inhibits iNOS expression, likely through bioactive phytochemicals such as phenolics, flavonoids, and terpenoids. These compounds may modulate pro-inflammatory signaling pathways, including NF-κB and MAPK, which regulate iNOS transcription (Surh et al., 2001; Kim et al., 1999). MYE reduced iNOS expression and NO production, likely by inhibiting NF-κB and MAPK signaling, thus limiting nitrosative stress while maintaining host defense. These results underscore MYE’s dual antioxidant and anti-inflammatory roles, supporting its therapeutic potential in managing intestinal inflammation and oxidative imbalance caused by the protozoan infection with *Eimeria* species (Bogdan, 2015; Shanmugasundaram et al., 2024).

Malondialdehyde (MDA) is a widely recognized marker of oxidative stress and a key byproduct of lipid peroxidation. In living systems, accumulation of ROS and free radicals creates a highly reactive environment that can damage cellular membrane lipids and proteins (Abdel-Latif et al., 2018). In this study, *E. labbeana*-like infection in pigeons significantly increased intestinal MDA levels and decreased GSH content compared to controls. Similar patterns have been observed in poultry models, where the infections with parasitic *Eimeria* species elevate lipid peroxidation markers while depleting key antioxidants, thereby disrupting redox balance (Santos et al., 2020; Tsiouris et al., 2021). Elevated MDA reflects oxidative damage to lipids and proteins critical for maintaining cellular membrane integrity, consistent with findings from Abdel-Latif et al. (2018) and Toah et al. (2021). Importantly, treatment with MYE significantly reduced MDA levels in infected pigeons, likely through direct antioxidant activity and indirect modulation of redox-sensitive signaling pathways such as NF-κB and MAPK, restoring cellular homeostasis and reducing inflammation. Plant-derived polyphenols and flavonoids are known to neutralize ROS and restore redox balance, reducing lipid peroxidation during coccidial infections (Tsiouris et al., 2021; Santos et al., 2020; El-Ghareeb et al., 2023). These findings underscore the complex interplay between *E. labbeana*-like infection, oxidative stress, and the protective potential of natural compounds like MYE. By mitigating oxidative damage, such interventions may help restore cellular homeostasis and support tissue protection during parasitic infections.

## Conclusion

It could be concluded that administering myrrh extract to pigeons infected with *E. labbeana*-like parasites may help reduce oxidative stress and intestinal damage. This approach offers potential advantages not only for the treatment and prevention of coccidiosis in pigeons but also as a promising natural strategy for improving intestinal health and disease resistance in commercial poultry systems. Further research is needed to isolate and characterize the bioactive constituents responsible for these effects and to confirm efficacy through larger-scale trials across different avian species. In addition, molecular and field-level studies are recommended to clarify the underlying mechanisms and evaluate the extract’s effectiveness under practical production conditions.

## Data Availability

The raw data supporting the conclusions of this article will be made available by the authors, without undue reservation.
